# Diseases and Injuries of Seamen, &c.

**Published:** 1855-03

**Authors:** 


### BOOK NOTICES.

*Diseases and Injuries of Seamen; with Remarks on their Enlistment, Naval Hygiene and the Duties of Naval Medical Officers.* By G. R. B. HORNER, M.D., Surgeon U. S. N.; Honorary Member of the Philadalphia Medical Society; Author of Medical Observations on the Mediterranean; The Medical Typography of Brazil and Uruguay, etc.

Nil actum reputans si quid superesset agendum.—LUC. LIB. ii. v. 657.

Philadelphia: Lippincott, Grambo & Co. 1854.

The above is the title of a volume of 252 pages, containing much valuable matter, in a plain and familiar style. The book is mostly the result of the author's own observations and experience during a period of twenty-eight years in the naval service.

Chapter I. treats of the duties of naval surgeons and apothecaries, and calls attention to the fact that merchantmen and emigrant ships are indifferently supplied with medical officers. The author recommends that an examining board be appointed at every important shipping port, for the purpose of testing the qualifications of those offering themselves for ship surgeons. It is justly remarked that, "so far as human life and comfort are concerned, it is just as important to have the best medical attendance in the merchant service as in the naval of any country."

Dr. H. gives us the mode of examination of recruits.

Candidates for entrance into the medical corps are also subject to an examination as to their physical abilities, and, in addition, are required to sign the following certificate:

"I declare upon my honor that my health at this time is good and robust, and to the best of my knowledge and belief I am free from constitutional defects, and without any phthisis, pulmonalis, gout, or chronic disease of any kind I have neither circocele, stricture of the urethra, hœmorrhoids, or hernia. Each and all of my organs of sense are without imperfection."

The following "General Order" may be of interest to some of our readers:

"Navy Department, Feb. 1, 1854.

"1. Hereafter a Board of Surgeons in the Navy shall assemble annually at such place as may be designated by the Department, about the close of the lecture sessions of the Colleges, for the examination and selection of candidates for admission into the Medical Corps of the Navy, and the examination of the Assistant Surgeons who may be candidates for promotion.

"2. The Board will select from the qualified candidates for admission, such a number of the best as may be necessary to meet the demands of the service for the following year.

"3. As vacancies occur in the Medical Corps of the Navy, appointments will be made from the qualified candidates in the order of succession in which they may be named by the Board; but no appointment will be given to any such candidate who is over 25 years of age.

"4. No qualified candidates will be held over for appointment after one year; but all such must be re-examined, and take position in the class in which they are last examined.

"5. Every candidate for admission will be examined, strictly and carefully, as to his physical capacity for the service; and the Board will make a separate report in each case, which will be forwarded *direct* to the Department, to be placed on file with the testimonials of the candidate. This examination will precede that as to professional qualifications, and no candidate who is not physically qualified will be examined professionally.

"6. In order that the relative position of Assistant Surgeons of the same date, who shall be examined for promotion at different times, may be more readily determined, a majority of the members of the Board will be selected, if practicable, from those who served on the next preceding Board.

"7. Assistant Surgeons, who are candidates for promotion, shall present to the Board testimonials of correct deportment and habits of industry from the Surgeons with whom they have been associated on duty; also, a Journal of Practice, or Case Book, in their own hand-writing. They are expected to be familiar with all the details of duty specified in the "Instructions for the government of Medical Officers."

Of 2128 persons examined in Philadelphia, 240, or more than 10 per cent, were rejected. Of these cases, sixteen were rejected for varicose veins of the legs, eleven for varicocele, twenty-seven for circocele on the left side, eighteen for myopia, nine for ophthalmia, six for blindness of the left eye, sixteen for imperfect forms, fourteen for various cutaneous eruptions, nine for diseased heart, ten for debility from old age, one for rupture in the right groin, and nine for rupture in the left groin.

The experience of Dr. H. in the treatment of syphilis and gonorrhœa is given in the following extract:

In the treatment of venereal ulcers, my uniform practice was to apply the lunar caustic once or more, then to heal them up with the black wash, or a solution of sulphate of copper or zinc, and if this failed or was not effectual enough, a little mercurial ointment was rubbed near the ulcers. Sometimes the blue pill was taken; in a few cases, when the ulcers sloughed, a solution of ʒi. or ʒii. of nitric acid in ℥viii. of water was applied with certain success. In phagedenic cases, this acid was occasionally used in its pure state, but as it is very severe undiluted, it should never be used if avoidable. The solution is preferable generally, and recently I have employed it oftener than the lunar caustic, as it does not stain the clothes or dressing, is milder, more conveniently used, and equal, if not superior, in healing power. After putting this solution over the chancre, it is brushed over with collodion, which soon dries, and proves a very fine dressing; but as it is apt to fall off from any accumulation of pus beneath, a dressing of oiled tissue-paper or adhesive plaster is then substituted.

If buboes followed the ulcers, they were if possible driven back by leeches or blisters, but these frequently failed, and the latter were objectionable from their inducing permanent induration of the skin as well as thickening of it. My practice, therefore, was generally to cause suppuration by applying linseed poultices.— But at Naples the virus of syphilis was so active and violent, that it quickly impregnated the system, acted like the poison of a viper on the parts, producing chancres and buboes in rapid succession, and I found that the best practice was to induce their absorption or suppuration by frictions with mercurial ointment in the course of the absorbents leading towards them. When constitutional symptoms followed they were combated with the syrup and decoction of sarsaparilla, and sometimes with blue mass in alterative doses. Other mercurial preparations were not employed at all, and salivation was never induced, yet very few instances of secondary syphilis happened, and no case was uncured, nor were any bones made carious, as under the old mercurial mode of treating syphilitic diseases, when patients were made to spit by the quart, according to John Hunter's practice. One of the most remarkable cases of secondary syphilis was that of an officer who contracted two chancres at Malta. They were healed with the nitrate of silver, black wash and lead water, without any apparent constitutional taint; no buboes formed, and yet six weeks after he was first treated for chancres, and some days after he returned to duty, a venereal ulcer formed in both tonsils. His diathesis was highly nervous and sanguine, the ulcers were irritable and inflamed, spread extensively, were very hard to cure and resisted the healing virtues of several gargles, as of borax, sage tea and honey, alone, or united with the tincture of myrrh. When these gargles had failed, a solution of five grains of the nitrate of silver to an ounce of water was substituted, and applied once or oftener every day with a mop. This medicine had the desired effect, and was gradually increased to ten grains, the patient was put on low diet, took five grains of blue mass every night, and a decoction of radix sarsaparillæ ℥iv., senna ℥i., extract of liquorice the same, extract of sarsaparilla ʒi., loaf sugar ℥viii., in a gallon and a half of water, which was boiled down a third. The next day after taking the blue pill he took a dose of powdered rhubarb. Afterwards some sassafras and mezereon were added to the decoction, but proved irritating to the throat, and were omitted. By the above treatment the ulcers were healed in about a month, but the fauces continued inflamed, and a scaly eruption appeared on the scalp, spread downwards over the back, and formed copper colored blotches over all the body and limbs. Not even the pœnis was exempted, and it became covered at the lower part with continuous scabs This eruption was as obstinate as the ulcers in the throat, which re-appeared in a short time, again healed up, and broke out again four months after they first appeared. The solution of nitrate of silver was perseveringly applied to them, and at last effected a cure, with the help of constitutional remedies. In addition to those named, hot bathing in fresh and sea water was used every day, seidlitz powders were substituted for rhubarb after the blue pill, and the skin kept perspirable by dissolving four grains of tartar emetic in a gallon of the diet drink. His condition was soon improved by this change of treatment, his throat and skin assumed their natural state, and he was restored permanently to duty early in August. His recovery was a subject of joy to himself, medical attendants and colleagues, who had been performing his duties so long, and would have probably been obliged to do so indefinitely, had he been treated according to the old routine, and thorougly mercurialized. For his irritable, sanguine temperament, and partly scrofulous habit, would certainly have been aggravated by such practice, and incurable ulcerations and caries been the probable consequences. His case illustrates, too, the very great importance of healing chancres immediately. His were not healed, I understand, from his living on shore when they broke out, and their having been allowed to attain a large size before the medicines mentioned were applied.

The style of the author is open to criticism; but the matter, much of it at least, must be of interest to the young practitioner, and especially to the naval surgeon. J.

